# Application of individual behavioral models to predict willingness to use modern contraceptives among pastoralist women in Afar region, Northern Ethiopia

**DOI:** 10.1371/journal.pone.0197366

**Published:** 2018-05-22

**Authors:** Znabu Hadush Kahsay, Dessie Tegegne, Ebrahim Mohammed, Getachew Kiros

**Affiliations:** 1 Health Education and Behavioral Science Unit, School of Public Health, Mekelle University, Mekelle, Ethiopia; 2 Department of Medical Laboratory Science, Debre Tabor University, Debre Tabor, Ethiopia; 3 Department of Public Health, College of Health Science, Samara University, Samara, Ethiopia; 4 Department of Health Education and Behavioral Sciences, Jimma University, Jimma, Ethiopia; Tulane University School of Public Health and Tropical Medicine, UNITED STATES

## Abstract

**Background:**

Use of modern contraceptive methods reduces the risk of unwanted pregnancy, and is influenced by individual-level factors. Willingness to use modern contraceptive methods maybe a useful metric when considering health outcomes as it could predict health behaviors. Therefore, the current study aimed to assess the willingness of women to use modern contraceptives in Afar pastoralist communities.

**Methods:**

A community-based cross-sectional study was conducted from May 1 to 30, 2016. Three hundred forty-five women of childbearing age (15–49 years) were systematically sampled with proportionate allocation from seven randomly selected *kebeles* (neighborhoods) in Aballa District of Afar Region, Ethiopia. All women meeting the inclusion criteria in each selected household were interviewed at home using a semi-structured questionnaire. Construct validity was assured using factor analysis. A combination of individual behavioral models were applied in order to measure willingness to use modern contraceptive methods. Multiple logistic regressions were utilized to identify factors associated with willingness to use contraceptive at P-value of less than 0.05.

**Results:**

Three hundred twenty-two women participated in the study, for a response rate of 93.3%. The mean age of respondents was 27 (±6) years. About one-third (N = 106, 32.9%) of the participants reported that they were willing to use modern contraceptives. Orthodox Christians (AOR = 4.22, 95% CI 1.94–8.92), women aged 19 or older at first marriage (AOR = 2.89, 95% CI 1.16–7.23), and women who had never experienced a stillbirth (AOR = 3.85, 95%CI 1.37–10.78) were more likely to report being willing to use modern contraceptives. Additionally, perceived severity of an unwanted pregnancy (AOR = 1.71, 95% CI 1.57–1.93) and perceived self-efficacy to use contraceptives (AOR = 1.26, 95% CI 1.17–1.65) were positively associated with the willingness. Women who had never had an abortion were less likely to express willingness to use modern contraceptives (AOR = 0.41, 95% CI 0.19–0.92) and perceived importance of cultural and religious norms (AOR = 0.85, 95% CI 0.62–0.90) was also negatively associated with willingness.

**Conclusions:**

The majority of women in this study were not willing to use modern contraceptive methods. A previous pregnancy outcome of stillbirth was associated with reduced willingness, while a prior abortion was associated with increased willingness. Perceived severity of unwanted pregnancy and higher self-efficacy surrounding contraceptive use were strong predictors of increased willingness to use contraceptives. Religious and cultural norms also appear to influence perception towards modern contraception. Thus, involvement of cultural and religious leaders and consideration of a woman’s reproductive history are recommended when designing health education messages on contraception for Afar pastoralist women.

## Background

A substantial number of women become pregnant every year without planning or desiring to increase their family size. Pregnancy-related deaths attribute up to 13% of the maternal deaths in the world [1]. Globally, about 40% of pregnancies in 2012 were unintended, and sub-Saharan countries contributed a considerable share to the proportion and its consequences [2]. Unwanted pregnancy often predisposes women to induced abortion. Out of 211 million estimated pregnancies occurring each year worldwide, about 46 million ends in an induced abortion [2]. Moreover, abortions in developing countries are often unsafe and performed by an untrained assistant in less-than-hygienic circumstances [2, 3].

Research findings indicate that modern contraceptive methods are effective in reducing the risk of unwanted pregnancy, reduce fertility rate and fertility-related challenges for women of childbearing age and their offspring. Contraceptives are also the most cost-effective strategy for reducing maternal and child mortality [4–6].

Despite potential to prevent unwanted pregnancy, modern contraceptive use is low among women of childbearing age in Ethiopia where it accounts for only 41% of the women [7]. The rate is lower in Ethiopia compared to other countries in Sub-Saharan such as in Mauritius (76%), Zimbabwe (60%) and South Africa (58%) [8]. Furthermore, the use of modern methods is much lower (11.6%), in rural regions such as Afar in Ethiopia, where use is more than three times lower than the national number [7].

Ethiopia is among countries with a high total fertility rate at 5.3 [7]. The country is the second most populous in Africa [9]. Nonetheless, about 22% of women of the reproductive age group in the country who want to delay or limit pregnancy do not actually use contraceptive currently. Meanwhile, the proportion is around 17.5% in Afar region [7]. This situation indicates that despite the Ethiopian government’s commitment to improving modern contraceptive use in the country in the past two decades [10, 11], the proportion of unmet need for modern contraceptive methods remains high in pastoralist communities of the country like Afar region.

Low modern contraceptive use is presumed to add to the health burden for the women of childbearing age in the Afar pastoralist community, in synergy with factors related to the nomadic lifestyle, high illiteracy levels among women, low access to health facilities, early child marriage, and the practice of female genital mutilation [12, 13]. Previous studies among pastoralist women in Eastern Africa also indicate that women’s individual level factors inhibit contraceptive use. Rural residence, low educational attainment, negative perception towards family planning, perceived oppositions from others (religious/cultural leaders and husband) are among the factors while women’s low decision-making power also correlates with low use of contraceptives [7, 14–20].

Even though low contraceptive use and high unmet need for modern contraceptives are evident in Afar region [7], there is a gap in the literature regarding the possible multi-level factors associated with low use. Therefore, the current study aimed to assess the willingness of women to use modern contraceptives, and associated factors among women of childbearing age in Abala district of Afar region, using individual behavioral models.

**Fig 1:**
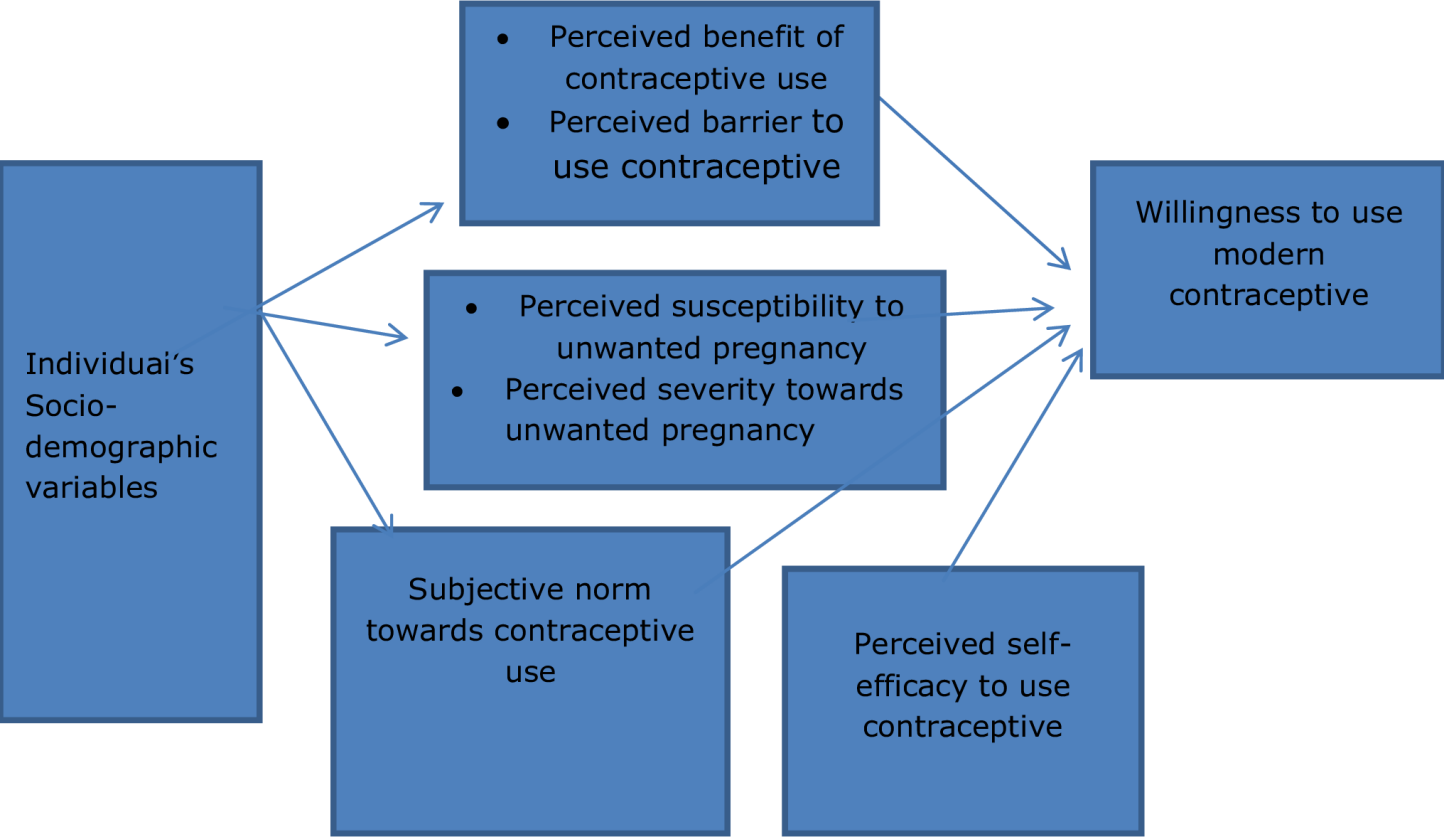
Theoretical framework for willingness to use modern contraceptive.

Evidence suggests that individual behavioral factors account for a notable share of factors preventing the use of modern contraceptives, whereas willingness to use these methods is a key. Application of behavioral models helps investigators to assess factors associated with service utilization behaviors in the perspective of the participants. It is also cited that there is no model or theory, which sufficiently explain the complexity of human health behavior while the predicting power of the models increases when complemented by constructs from other models [21–22]. Thus, the investigators adopted subjective norm from Theory of planned behavior (TPB) to supplement the limitation of Health Belief Model (HBM) to capture the role of approval or disapproval of referents for recommended behavior such as contraceptive use. Consequently, the current study adopted all constructs of the Health Belief Model (HBM) and subjective norm from Theory of Planned Behavior (TPB) for prediction of individual-level factors for willingness to use modern contraceptives. As Fig 1 shows, the constructs postulate that willingness to use modern contraceptives is determined by the individual’s perceived threat (perceived susceptibility and perceived severity) towards unwanted pregnancy, their perceived benefit towards modern contraceptive use, their perceived barrier to use of the methods, their perceived self-efficacy, and subjective norms related to use of modern contraceptive methods [21–24].

## Methods and materials

### Study area

Aballa district is one of the districts in KilbetRasu zone of Afar regional state in Ethiopia. It is the administrative center of the zone. It is located 775 Km North of Addis Ababa, the capital of Ethiopia, and 489 Km North West of Samara town, the Capital of Afar regional state. Based on figures from the Central Statistical Agency in 2005, the projected total population for Abala district is 6601 of which 3153 of them are women by the year of 2016[25]. The district constituted 14 kebeles (Smallest administrative unit).

The region is also characterized by a high fertility rate (5.7), low modern contraceptive use (11.6%) and low access of health services like antenatal care (50%) and low utilization of skilled birth attendants (15%) [7]. Afar region contains the highest proportion of a regional population within a lower wealth quintile (60%) in the country. Pastoralists of the area, like pastoralists in other settings, remain at the margins of national economic and political life, and pastoral women are vulnerable to poor health outcomes. Pastoralists depend heavily on livestock products, which are highly dependent on the availability of water.

### Study design and study period

A community-based cross-sectional study was conducted among women of childbearing age (15–49) in Aballa district of Afar region, from 1st to 30th May 2016.

### Sample size determination and sampling procedure

The sample size was calculated using a single population proportion formula, applying the following parameters: proportion of women who use modern contraceptive in Afar region, which is 8.4% (26), at 95% Confidence interval(CI), assuming 5% level of significance and 3% allowance of margin of error (d = 0.03). Therefore, the minimum Sample size required (n) was calculated as;

$$n={\left(Z\alpha /2\right)}^{2}\;*P\;\left(1‑P\right)/d^{2}=1.96^{2}\;*\;0.084\left(0.916\right)/{\left(0.03\right)}^{2}=328$$

To compensate for possible non-response rate, 5% was added to the sample size. Finally, the minimum sample size required was 345 participants.

From 14 kebeles (the smallest administrative unit in the district), seven were selected randomly using lottery method. A total number of estimated women aged 15 to 49 was computed for each selected kebele using population from the National Central Statistical Agency (CSA) in 2007 [25]. Then the sample size for each of the selected kebeles was determined according to population proportional to size method. Then, systematic random sampling was employed to select the households with eligible women using the kebele health extension worker’s updated sampling frame for women of childbearing age (15–49). Women aged 15 to 49 years residing in selected households were interviewed. Whenever two or more than two eligible women were found in the household, one was selected using lottery method. Among eligible women, those who were not able to communicate due to serious illness were excluded.

### Data collection instruments and procedure

The data collection instrument (See S1 File) was developed in English from related literature [15, 26, 27, 28] and contextualized for the current study objectives. The tool was translated into local language by language expert and back-translated by another expert. It consisted of three main parts: socio-demographic and obstetric information of the respondents; six constructs of the behavioral models and willingness to use contraceptives. The constructs measured were (a) perceived susceptibility to unwanted pregnancy; (b) perceived severity of unwanted pregnancy; (c) perceived barriers to use of modern contraceptives; (d) perceived benefits of modern contraceptive use; (e) perceived self-efficacy to use the methods; and (f) subjective norms. Finally, an item which measures willingness to use modern contraceptive methods was included.

Six female diploma-graduate nurses who know the local culture, and speak the local language, collected the data. They approached study participants at home for interviews using the pretested interviewer-administered questionnaire.

### Data quality control

To assess the reliability of the tool, questionnaire test-retest was done in two-week intervals among participants in similar kebeles, but outside of the kebeles included in the study. Its interaclass correlation Coefficient (ICC) was found to be 0.8, indicating an acceptable value for the reliability of the tool. Then, the questionnaire was pre-tested on 5% of the sample size in a similar setting. The investigators trained data collectors for three days on objectives and processes of the study. Three supervisors followed the overall activities of data collection on a daily basis. Principal investigators checked at least thirty percent of the filled questionnaires daily for completeness and accuracy. Questionnaires with incomplete or inconsistent response were omitted. Double data entry was used to minimize errors. Finally, factor analysis was done to ascertain construct validity, and item reliability for each construct was found to be acceptable (using Cronbach’s alpha >0.7).

### Measurement

Constructs of the model were measured by items in five-point Likert scale that ranges from strongly agree (5) to strongly disagree (1). Negatively worded statements were reverse coded before analysis. Then, 32 items were subjected to factor analysis (using principal components method, Varimax rotation method, and a fixed number of factors) to extract the relevant valid constructs.

The output of the factor analyses indicated that the constructs explained 63.1% of the variance in willingness to use modern contraceptives cumulatively. With respect to the number of items loaded to the constructs, eight items were loaded to a perceived threat [e.g. I am at risk of having unwanted pregnancy, If I do not use contraceptive], seven items to perceived benefit [e.g. Modern contraceptive use can prevent risk of unwanted pregnancy] and four items to a perceived barrier [e.g. It is too far to travel for me to get modern contraceptive methods]. The other constructs consisted of three items in the factor loading. After valid constructs were identified, reliability testing of items in the respective constructs was assessed and found to be acceptable. Then, the score of for each item summed up to produce a composite score, which was used for further analysis.

#### Outcome variable

Among the respondents who ever had sexual intercourse in the 12 months prior to the study, participants were asked about willingness to use modern contraceptives in the future to prevent pregnancy.

#### Data analysis

The data were entered into Epi Data version 3.1 then exported to SPSS for Windows version 20.0. Descriptive statistics were used to summarize the data. Binary logistic regression was done to identify factors that were significantly associated with being willing to use modern contraceptives. All variables which were significant on bivariate analysis (p < 0.05) were entered into multiple logistic regressions models. The odds ratio at 95% confidence interval was reported to declare significant association.

### Ethics approval and consent to participate

Ethical approval to conduct the study was granted by the Ethical Review Board of Samara University. The board waived the requirement for parental consent for the study. The board also wrote a formal letter of cooperation to the district’s health office where the study was conducted. Study participants were informed about the objectives of the study, assured for confidentiality. Then, written informed consent was obtained from each participant.

## Results

### Socio-demographic characteristics of respondents

In total, 322 respondents participated in this study producing a response rate of 93.3%. All of the participants reported that they were sexually active in the 12 months prior to the study. Of the respondents, only 12.1% were current users of modern contraceptives, and 17.3% have ever used them. Moreover, 32.9% of them reported that they were willing to use modern contraceptives to prevent pregnancy while the remaining 216 (67.1%) did not report willingness.

The mean age of respondents was 27 (SD = 6). The majority (84.2%) of the respondents were currently married, of which 72.6% married at age 18 years and below. About 88.2% of the respondents described their occupation as homemaker/housewife and 78.3% were illiterate (unable to read and write). The majority (95.7%) them were Muslim in religion, and about 36.0% of respondents reported an average of 400 and below Ethiopian birr (17 USD) as their income level per month.

Nearly forty percent of the respondents reported an experience of unplanned pregnancy, and 27.6% experience of unsafe abortion (illustrated in Table 1).

**Table 1:** Demographic and obstetric characteristics of respondents in Abala district, Afar region of Northern Eastern Ethiopia 2014.

Variables	Categories	Total Number (percentage)
Age	< = 29	109(33.9)
	30–34	100(31.1)
	> = 35	113(35.1)
Marital Status	Currently Married	271(84.2)
	Currently not married	51(15.8)
	Total	328(100)
Occupational Status	House Wife	284(88.2)
	Farmers or Merchants	38(11.8)
Educational Status	Illiterate	252(78.3)
	Literate	70(21.7)
Residence	Rural	237(73.6)
	Urban	85(26.4)
Religion	Muslim	308(95.7)
	Others*	14(4.3)
Ethnicity	Afar	267(82.9)
	Others**	55(17.1)
Age at first Marriage	< = 18	220(72.6)
	> = 19	83(27.3)
Age at First Pregnancy	< = 18	143(48.7)
	> = 19	151(51.3)
Ever use for Traditional contraceptive methods	Rhythm	32(64.0)
	Withdrawal	18(36.0)
Ever had Unplanned Pregnancy	Yes	127(39.4)
	No	193(59.9)
Ever had Abortion	Yes	86(27.6)
	No	226(72.4)
Ever had still birth	Yes	45(13.9)
	No	277(85.1)
Total		322(100)

* = Orthodox and protestant

** Tigre and Amhara

### Factors predicting willingness to use modern contraceptives

To examine the statistically significant factors associated with willingness to use modern contraceptives, multiple logistic regressions was utilized. The fitness of the model to predict the probability of willingness to use modern contraceptives in pastoralists was assessed and found to be statistically significant [-2loglikelyhood = 231.808, x^2^ = 109.749, degree of freedom = 11 with a p-value<0.0001 with the overall prediction of 80.5%].

Results indicated that religion, age at first marriage, an experience of stillbirth, and experience of abortion were significantly associated with the likelihood of being willing to use modern contraceptives. Meanwhile, perceived severity, perceived benefit, and self-efficacy were positively associated with willingness to use modern contraceptive while perceived partner norms and perceived cultural and religious norms were negatively associated in the bivariate logistic regression (Table 2).

**Table 2:** Factors associated with willingness to use modern contraceptives, in bivariate logistic regression, among pastoralist women of childbearing age (15–49) Afar, Ethiopia, 2014.

Variables	Categories	Willingness to use contraceptives		COR(95% CI)
		Not willing	Willing	
Age Group	< = 29	69(63.3%)	40(36.7%)	1
	30–34	63(63.0%)	37(37.0%)	1.01 (0.58–1.77)
	> = 35	84(74.3%)	29(25.7%)	0.60(0.34–1.06)
Marital Status	Married	193(71.2%)	78(28.8%)	1
	Currently not married	23(45.1%)	28(54.9%)	3.01(1.64–5.55)
Occupational Status	House Wife	203(71.5%)	81(28.5%)	1
	Farmers or merchants	13(34.2%)	25(65.8%)	4.82(2.35–9.88)
Religion	Muslim	209(67.9%)	99(32.1%)	1
	Orthodox	7(50.0%)	7(50.0%)	2.11(0.72–6.18)
Experience of still birth	Yes	36(80.0%)	9(20.0%)	1
	No	172(65.2%)	92(34.8%)	0.56(0.35–0.90)
Age at first marriage	< = 18	150(65.5%)	79(34.5%)	1
	> = 19	66(71.0%)	27(29.0%)	0.56(0.35–0.90)
Experience of abortion	Yes	56(65.1%)	30(34.9%)	1
	No	153(67.7%)	73(32.3%)	0.89(0.53–1.50)
Perceived Severity	Mean	B = 0.120		2.12(1.86–2.49)
Perceived Benefit	Mean	B = 0.134		1.14(1.04–1.26)
Perceived Partner’s norm	Mean	B = -0.223		0.80(0.68–0.94)
Perceived cultural and religious norm	Mean	B = -0.155		0.85(0.74–0.98)
Self-efficacy	Mean	B = 0.141		1.86(1.17–2.16)

The outputs of Multiple Logistic Regressions indicated that the probability of being willing to use modern contraceptive among orthodox Christian women was 4.22 times more likely than that for Muslim women (AOR = 4.22, 95% CI = (1.94–8.92)). Meanwhile, the likelihood of being willing to use modern contraceptives was 2.89 times more likely among respondents married at age of 19 and above, compared with those who married at age 18 and below (AOR = 2.89, 95% CI = (1.16–7.23)). Additionally, respondents with no experience of stillbirth were 3.85 times more likely to be willing to use modern contraceptives than the respondents with no history of stillbirth in their life time (AOR = 3.85, 95% CI = (1.37–10.78) (Table 3).

**Table 3:** Factors associated with willingness to use modern contraceptives, in multiple logistic regression, among pastoralist women of childbearing age (15–49) Afar, Ethiopia, 2014.

Variables	categories	Willingness to use		AOR(95% CI)
		Willing	Not willing	
Religion	Muslim	209(67.9%)	99(32.1%)	1
	Orthodox	7(50.0%)	7(50.0%)	4.22(1.94–8.92)
Experience of Still birth	Yes	36(80.0%)	9(20.0%)	1
	No	172(65.2%)	92(34.8%)	3.85(1.37–10.78)
Age at first marriage	< = 18	150(65.5%)	79(34.5%)	1
	> = 19	66(71.0%)	27(29.0%)	2.89(1.16–7.23)
Experience of abortion	Yes	56(65.1%)	30(34.9%)	1
	No	153(67.7%)	73(32.3%)	0.41(0.19–0.92)
Perceived Severity towards unwanted pregnancy	Mean	B = 0.159		1.71 (1.57–1.93)
Perceived cultural and religious norm	Mean	B = -0.156		0.85 (0.62–0.90)
Self-efficacy	Mean	B = 0.60		1.26(1.17–1.65)

Moreover, the likelihood of being willing to use modern contraceptives was positively associated with increasing score in respondents’ perceived severity towards unwanted pregnancy and perceived self-efficacy to use contraceptives. However, it was negatively associated with respondent’s score forperceived cultural and religious norms to use contraceptives. As shown in Table 3, a unit change in respondent’s score for perceived severity of unwanted pregnancy increased the odds of being willing to use modern contraceptives by 71% (AOR = 1.71,(95%CI = (1.571–1.93)). Likewise, a unit change in score of perceived self-efficacy also increased the likelihood of being willing to use modern contraceptive by 26% (AOR = 1.26 (95%CI = (1.17–1.65)). As with a subjective norm, a unit increase in perceived cultural and religious norm decreased the odds of being willing to use modern contraceptives by 85% (AOR = 0.85, 95%CI = (0.62–0.90)).

## Discussion

The study aimed to determine the willingness of pastoralist women to use modern contraceptives and to identify factors associated with willingness. The current study indicated that only one out of three (33%) of respondents reported a willingness to use modern contraceptives in their future to prevent pregnancy. The proportion of current users (12%) was much lower than the national modern contraceptive use rate of 41% [7]. However, it was slightly higher than from that of the Mini Ethiopian Demographic Health Survey (MDEHS) report for Afar region, at 9.5% [26] and previous study in the region [13]. This may indicate that a lag in the proportion of modern contraceptive users in Afar pastoralist women.

Regarding religion, Muslim participants were less likely to be willing to use modern contraceptives compared to non-Muslim participants. Furthermore, increasing score for perceived disapproval from cultural and religious norms was negatively associated with willingness to use modern contraceptives. These findings were in tandem with previous studies that highlight the important role of religious and cultural factors [27, 28,29] in modern contraceptive because women residing in the pastoral community tend to comply with the religious believes to avoid disapprovals. A secondary analysis of Ethiopian Demographic Data (DHS) also yields that Muslim follower women were less likely to use modern contraceptives [15]. Previous studies in Afar and Bale pastoralist communities of Ethiopia also showed low willingness to use modern contraceptives and more than half of the women put the fear of religious disapproval as reasons or contraceptive non-use. Furthermore, previous studies from Afar [20] and Bale eco-region of Ethiopia [14] also reveal women’s perception of cultural acceptability predicts contraceptive use. A similar finding was also reported from Somalia refugees in Kenya [27].

This may imply that women tend to be less willing to use the contraceptives where fear of disapproval from the cultural and religious leaders in their community is a factor. According to Ajzen’s Theory of Planned Behavior (TBP), intention/willingness to commence health behavior, e.g. contraceptive use, is predicted by the function of referents’ (e.g. religious and cultural leaders) approval towards the behavior and the individual’s motivation to comply with the referents suggestion [24]. However, parent disapproval was not significantly associated with willingness to use contraceptives, which was dissimilar with previous findings from Afar and Kenya pastoralist women [20, 28]. This may give a clue that women’s fear regarding their husband’s disapproval might get lighten with the existed interventions directed to awareness creation in the country.

In congruence with evidence stated above, the current study also revealed that women’s perceived threat towards unwanted pregnancy was a statistically significant factor for being willing to use contraceptives. This was consistent with one of the premises of Health Belief Model (HBM), which presumed the likelihood of individual’s health behavior is predicted by individuals’ perception regarding the personalized risk of the problem/s and the severity of the sequels [21, 23].

Regarding obstetric history, odds of being willing to use modern contraceptives among women who had no experience of abortion were lower than those for their counterparts. This may indicate that the consequences of post-abortion experience may lead pastoralist women to favor modern contraceptive use, to avoid the possible risk of unwanted pregnancy. This was in line with recent studies from Ethiopia that reveal higher contraceptive use rate among women who ever experienced abortion [30, 31]. Following the experience of abortion, women get motivated to use modern contraceptives to avoid the risk of unwanted pregnancy [32]. The current study also showed that women with no experience of stillbirth were more likely to be willing to use modern contraceptives, which is in line with the review of Ethiopian Demographic Health Survey (EDHS) report. The review shows that child mortality has a significant inverse relationship with contraceptive use and one’s own child’s death is associated with the negative influence of contraceptive use [15]. A qualitative study among Somalia refugees recognized the history of child loss prevents women to use contraceptive [27]. In the current study, 14% of the women ever experience stillbirth. A study in Bale pastoralist community also reported slightly higher proportion (19%) of women ever had child loss [14]. Thus, in combination with other negative perceptions and misconceptions held by pastoralist women [29], child loss and stillbirth may predispose low willingness to use a modern contraceptive.

The current study also revealed increasing age at first marriage was positively associated with the probability of being willing to use modern contraceptives. This result was congruent with the 2012 Ethiopian Demographic health survey report that revealed lower contraceptive use among women aged less than 19 years than women aged 19 and above [33]. This may indicate that younger women might not consider themselves at risk of unwanted pregnancy or unaware of potential consequences. Content and approach to awareness creation activities provided in pastoralist community could potentially be tailored to personalize the risks of unintended pregnancy.

Perceived self-efficacy of women to use modern contraceptives was positively associated with being willing to use a modern contraceptive. This finding is consistent with previous studies done in Kenya and Nigeria [34] as well as in Uganda [35], which both indicate perceived self-efficacy significantly predicts modern contraceptive use [35]. Other previous studies also document that women’s decision making power to use maternal health services, including contraceptive use, is low in pastoralist community [17,18,36]. For the most part of pastoralist community, women do control less resource and rarely decide independently in health matters. Consequently, the women’s perception of their power to decide independently may lead them to be less willing to use contraceptives.

### Limitations of the study

As this study aimed at identifying individual-level behavioral factors, considering other social factors may also be needed to implement the findings of the current study.

### Conclusions and recommendations

In the study area willingness to use modern contraceptive was low among pastoralist women. Furthermore, those who were willing to use the methods were not currently using them. Women’s individual behavioral factors (specifically, perceived negative consequences of unwanted pregnancy, perceived self-efficacy to use the modern contraceptives, and perceived disapproval from cultural and religious norms) were predictors of willingness to use modern contraceptives. Hence, the current study suggests that design and implementation of tailored health education interventions to improve women’s perception of the possible risks and consequences of an unwanted pregnancy may improve uptake of modern contraceptives. In addition, ensuring involvement of cultural and religious leaders in awareness creation are also recommended.

## Supporting information

S1 File English questionnaire. (DOCX)
