# Supplementary material for: Application of individual behavioral models to predict willingness to use modern contraceptives among pastoralist women in Afar region, Northern Ethiopia
Source: PLoS One. 2018 May 22;13(5):e0197366. doi: 10.1371/journal.pone.0197366 (PMC5963766; doi:10.1371/journal.pone.0197366)
Supplement: S1 File — (DOCX) [file pone.0197366.s001.docx]

S1. English questionnaire

**Informed Consent**

Hello, my name is _________________ and I am from Samara university and I am currently carrying out community cross-sectional survey on predictors of modern contraceptive utilization using behavioral models among women of child bearing age, in Abala district of afar region, in scientifically, sampled enumeration kebeles. As part of this survey I am collecting information from pregnant mothers in the sampled households by conducting interview.

This interview will be analyzed with high confidentiality; personal identification will not be needed. May I now ask that you to participate in this survey? However, if youdecide either not to have the interview done or to discontinue the interview, it is your right and i will respect your decision. Now please tell meif you agree to have the interview done.

Yes__________ no ____________

Signature of interviewer ___________________________

Questionnaire

1. **Residence** Rural Urban
2. **Age (in years)**
3. **Religion** Muslim Orthodox Protestant Other(specify)______________
4. **Ethnicity** Afar AmharaTgrie other(specify)_____________
5. **Educational status** Can not read and write Can read and write ( greade-------)
6. **Occupational status** House wife Farmer Employed Merchant Others ( specify) _____________
7. **Marital status** Single married divorced widowed
8. Average monthly income in birr -----------

*Obstetric characteristics of the respondents*

1. Age during marriage ------------
2. Age at first pregnancy ----------
3. Number of all pregnancies ----------
4. Family size ----------
5. Length of time between the past two successive live births if so _________
6. Ever had unplanned pregnancy? Yes No
7. Ever had history abortion? Yes No
8. if " Yes" for Q14 was it: Induced Spontaneous
9. history of still birth Yes No
10. history of neonatal death Yes No
11. Ever had complication during pregnancy ? Yes no i don’t remember

**knowledge**

1. Do you know any traditional method of contraceptive? Yes No
2. If yes for Q20 which one

Rhythm Withdrawal Other

1. Do you know any method of modern contraceptive?
2. If yes for Q21 which one

Pill Injectables IUD Diaphragm Female sterilization Male sterilization Any other(specify)_______________

**Perceived susceptibility**

1. I am at risk of unwanted pregnancy if I don't use contraceptive?

1) strongly disagree 2) disagree 3) I don't know 4)agree 5) strongly agree

1. I am at risk of practicingabortion if I don't use contraceptive?

1) strongly disagree 2) disagree 3) I don't know 4)agree 5) strongly agree

1. I am at risk of developing HIV/AIDS during unsafe abortiondue to unclean materials

1) strongly disagree 2) disagree 3) I don't know 4)agree 5) strongly agree

1. There is risk of maternal death if unwanted pregnancy happen ?

1) strongly disagree 2) disagree 3) I don't know 4)agree 5) strongly agree

1. I am at risk of having extended family if I don't use contraceptive??

1) strongly disagree 2) disagree 3) I don't know 4)agree 5) strongly agree

**Perceived severity**

1. unwanted pregnancy can resultin unsafe abortion

1) strongly disagree 2) disagree 3) I don't know 4)agree 5) strongly agree

1. unsafe abortioncan lead to maternal death

1) strongly disagree 2) disagree 3) I don't know 4)agree 5) strongly agree

1. unwanted pregnancycan affect a family economically .

1) strongly disagree 2) disagree 3) I don't know 4)agree 5) strongly agree

1. Births with close gap can affect the health of a woman

1) strongly disagree 2) disagree 3) I don't know 4)agree 5) strongly agree

1. Births with close gap can affect the health of chilren

1) strongly disagree 2) disagree 3) I don't know 4)agree 5) strongly agree

**Perceived benefit**

1. Modern contraceptives can prevent pregnancy effectively

1) strongly disagree 2) disagree 3) I don't know 4)agree 5) strongly agree

1. Modern contraceptives prevents abortion related consequences

1) strongly disagree 2) disagree 3) I don't know 4)agree 5) strongly agree

1. Modern contraceptives helps in birth spacing

1) strongly disagree 2) disagree 3) I don't know 4)agree 5) strongly agree

1. Modern contraceptives helps in permanently stop fertility if wanted.

1) strongly disagree 2) disagree 3) I don't know 4)agree 5) strongly agree

1. Some modern contraceptives can prevent HIV/AIDS transmission .

1) strongly disagree 2) disagree 3) I don't know 4)agree 5) strongly agree

1. Other benefit if there (specify) ________________________

**Perceived barrier**

1. It is too far to get modern contraceptives to me?

1) strongly disagree 2) disagree 3) I don't know 4)agree 5) strongly agree

1. I fear side effects to use modern contraceptives

1) strongly disagree 2) disagree 3) I don't know 4)agree 5) strongly agree

1. Cost is not affordable to me to use modern contraceptive

1) strongly disagree 2) disagree 3) I don't know 4)agree 5) strongly agree

1. It is inconvenient for me to use modern contraceptives

1) strongly disagree 2) disagree 3) I don't know 4)agree 5) strongly agree

1. Other barrier(specify)________________

**Subjective Norms**

1. It is against my religious norm to use modern contraceptives

1) strongly disagree 2) disagree 3) I don't know 4)agree 5) strongly agree

1. It is against my cultural norm to use modern contraceptives

1) strongly disagree 2) disagree 3) I don't know 4)agree 5) strongly agree

1. My husband will not acceptance to use modern contraceptives .

1) strongly disagree 2) disagree 3) I don't know 4)agree 5) strongly agree

1. My peers will discriminate me if they know I am using modern contraceptives

1) strongly disagree 2) disagree 3) I don't know 4)agree 5) strongly agree

1) strongly disagree 2) disagree 3) I don't know 4)agree 5) strongly agree

**Perceived self efficacy**

1. I feel confident that modern contraceptive will prevent unwanted pregnancy

1) strongly disagree 2) disagree 3) I don't know 4)agree 5) strongly agree

1. I would have a confidence to suggest my partner to use modern contraceptive

1) strongly disagree 2) disagree 3) I don't know 4)agree 5) strongly agree

1. I am confident to ask modern contraceptive methods in health institution .

1) strongly disagree 2) disagree 3) I don't know 4)agree 5) strongly agree

1. I feelthe cost for modern contraceptive is easyto overcome .

1) strongly disagree 2) disagree 3) I don't know 4)agree 5) strongly agree

**Utilization**

1. Have you ever used any contraceptive Yes NO
2. Haveever do sex in the past 12 months Yes NO
3. Are you currently using any contraceptive in the past 12 months Yes No
4. If yes for question “55’’ which one do you use

1.pill

2.Depo/injectablle

3.Implanon

4.diaphram

5.vasicotomi

6.tuiubaligation

7.IUCD

8.other/specify____________

1. If yes from what do you hear about the method.

1.health professionals

2.peer

3.radio.

4.TV

5.poster

6.other (speify)_________________

***I Would Like To Thank You For Your Patience In Completing This Questionnaire!!!***
